# *IAT4*, a New Indolamine *N*‐Acetyltransferase in *Saccharomyces cerevisiae* Involved in Melatonin Biosynthesis

**DOI:** 10.1111/jpi.70053

**Published:** 2025-05-09

**Authors:** Andrés Planells‐Cárcel, Sandra Sánchez‐Martí, Sara Muñiz‐Calvo, José Manuel Guillamon

**Affiliations:** ^1^ Departamento de Biotecnología de Alimentos, Instituto de Agroquímica y Tecnología de Alimentos (IATA) Consejo Superior de Investigaciones Científicas (CSIC) Valencia Spain; ^2^ Chalmers University of Technology Gothenburg Sweden

**Keywords:** 5‐methoxytryptamine, melatonin, *S. cerevisiae*, serotonin, serotonin *N‐*acetyltransferase

## Abstract

Melatonin synthesis by yeast has been described on several occasions, mainly in a fermentative context. However, the genetic determinants involved in its synthesis remain undefined. Understanding melatonin synthesis in yeast is important because it can provide insights into the broader mechanisms of indolamine production, which has implications for both basic biological research and industrial applications. Although two genes with *N*‐acetyltransferase (NAT) activity (*PAA1* and *HPA2*) have been identified in *Saccharomyces cerevisiae*, these genes do not seem to be major contributors to the production of melatonin and other indolamines in yeast in vivo. In this study, we identified the uncharacterized gene YDR391C as the gene encoding a protein with NAT activity, herein named *IAT4*. By comparing different substrates using the purified Iat4, we found that the *K*_*m*_ values were 353, 356, and 930 µM towards 5‐methoxytryptamine, tryptamine, and serotonin, respectively. The substrate affinity of Iat4 towards serotonin was approximately five times higher than that reported for the previous homolog of the melatonin enzyme arylalkylamine *N*‐acetyltransferase (*PAA1*), suggesting that *IAT4* could play a more significant role in melatonin biosynthesis. This enhanced affinity could lead to more efficient production of *N*‐acetylserotonin, potentially improving yields in biotechnological applications. Finally, we demonstrate the conversion of serotonin into microbially‐produced *N*‐acetylserotonin by overexpressing *IAT4* in a serotonin‐overproducing yeast strain at a titer of 14.5 mg/L. These findings represent the first steps towards the development of yeast strains optimized for the biological production of *N*‐acetylserotonin and related compounds, which might aid in studying the regulatory mechanisms and functions related to melatonin biosynthesis in *S. cerevisiae* and other yeast species.

## 1 Introduction

Melatonin appears to be a universal molecule present in most organisms [1, 2]. It was initially discovered in the pineal glands of vertebrates [3, 4], especially mammals, as a molecule involved in the regulation of the circadian cycle [5]. But further research has shown its presence in other pluricellular organisms such as invertebrates [6] and plants [7], even in monocellular organisms such as bacteria, cyanobacteria [8, 9], and yeasts [10].

Melatonin shows functional roles among organisms. In animals, melatonin is primarily a neurohormone, participating in the regulation of circadian rhythm. It has several other physiological functions such as modulating the immune system, protection against UV light, and protection against ageing [11, 12, 13]. In plants, it has been involved as a signal molecule in multiple mechanisms such as plant development and growth, besides as an antioxidant effect and resistance against various stresses [14].

In the case of the yeast *Saccharomyces cerevisiae*, a protective role against oxidative stress, such as UV light and oxidising agents, has been demonstrated [15, 16, 17]. Melatonin was described in yeast for the first time by Sprenger et al. [10] as a product of the metabolism of tryptophan. Subsequently, it has been studied for its emergence and involvement in fermentation processes like wine, beer, and other fermented products [18, 19, 20, 21]. Recently, it has been discovered to be involved in the binding to glycolytic proteins in strains with high fermentative capacity [22, 23].

**Figure 1:**
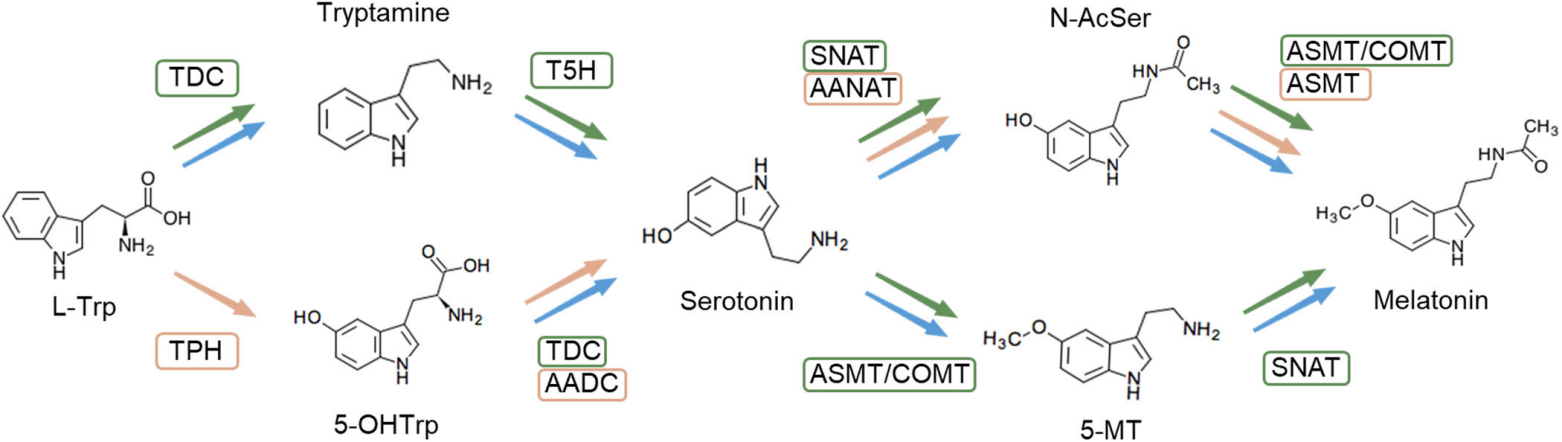
Illustration of the biosynthetic pathway of melatonin from tryptophan for different organisms. 5‐OHTrp, 5‐hydroxytryptophan; 5‐MT, 5‐methoxytryptamine; l‐Trp, tryptophan; NAcSer, *N*‐acetylserotonin. Enzymes represented are AADC, aromatic amino acid decarboxylase; AANAT, aromatic amino acid *N*‐acetyltransferase; ASMT, *N*‐acetylserotonin methyltransferase; COMT, caffeic acid *O*‐methyltransferase; SNAT, serotonin *N*‐acetyltransferase; T5H, tryptamine 5‐hydroxylase; TDC, tryptophan decarboxylase; TPH, tryptophan hydroxylase. Colours refer to the pathway and enzymes usually employed by each organism. Orange refers to mammalian pathway, green for plants, and blue for yeast.

Melatonin synthesis pathway starts from the amino acid tryptophan and involves 4 enzymatic activities: decarboxylation, hydroxylation, *N*‐acetylation, and *O*‐methylation. These activities seem to be conserved in all organisms, but they differ in the order in which these reactions are performed [1, 24] (Figure 1). In the case of *S. cerevisiae*, Muñiz‐Calvo et al. [24] elucidated which would be the preferential enzymatic pathway in yeast, resulting in a hybrid pathway between the mammalian and plant pathways. This indicates that tryptophan is first decarboxylated to tryptamine, followed by hydroxylation to form serotonin. Both pathways are then possible to reach melatonin, either *N*‐acetylation to *N*‐acetylserotonin followed by *O*‐methylation to give melatonin, or *O*‐methylation of serotonin to give 5‐methoxytryptamine followed by *N*‐acetylation to give melatonin.

Currently, there is a lack of information on which gene determinants are implicated in this synthesis pathway in yeast. The only gene described and characterised in the literature is *PAA1*, a polyamine acetyltransferase, homolog of the vertebrate's aralkylamine *N*‐acetyltransferase (*AANAT*) that is able to acetylate serotonin to *N*‐acetylserotonin and 5‐methoxytryptamine to melatonin [25]. However, further research has shown that *PAA1* is not primarily involved in the *N*‐acetylation of aralkylamines, instead it is involved in acetylation of polyamines, such as putrescine, spermidine, and spermine [26]. Recently, we also confirmed that the aralkylamine *N*‐acetyltransferase activity does not appear to be the main acetylating activity of Paa1 [27]. We also showed that there are other enzymes, such as *HPA2*, that can take over the function and provide this activity when *PAA1* is deleted [27]. In this previous study, we also pointed to the existence of other indole *N*‐acetyltransferases specifically involved in the melatonin biosynthetic pathway. Thus, the search for new candidate *N*‐acetyltransferases involved in melatonin synthesis remains a challenge.

Traditionally, sequence‐based searches using tools such as the Basic Local Alignment Search Tool (BLAST) [28] have been widely used to identify homologous genes. Although computational tools that predict protein structures from known templates have been available for several decades, current bioinformatics tools include structural domains and tertiary structure to find proteins that, despite differences in amino acid sequence, produce similar structures with the same enzymatic function. This allows the identification of functionally equivalent enzymes that might be missed by sequence‐based methods alone. Examples of these servers are Foldseek or DALI, which align protein structures against large protein structure collections [29, 30]. Complementary to these servers, advances in machine learning in recent years have led to the appearance of the Alphafold database, a collection of thousands of predicted protein structures [31], which has meant an exponential increase in the number of structures that can be used in these servers. In addition, computer simulations of substrate–protein interactions provide a rapid approach to identifying candidate enzymes for a desired enzymatic reaction of study and have been widely used for virtual drug screening [32]. The molecular docking technique allows the calculation of the affinity of an enzyme for a substrate, the evaluation of possible interactions between the substrate and the functional groups of the amino acids that make up the enzyme, and can even help to identify the binding mode of catalysis [33]. These advances have made it possible to generate a new approach in the search for homologous proteins based on their structure, opening the door to finding new proteins that are candidates for homologous functions in those pathways that have yet to be discovered.

In the present study, the use of Foldseek was applied to discover a novel indole *N*‐acetyltransferase enzyme that utilizes metabolites involved in the melatonin synthesis pathway as substrates. In this search, the gene of unknown function YDR391C, herein named *IAT4*, was identified as a putative indolamine *N*‐acetyltransferase. This enzyme exhibits catalytic activity similar to the aralkylamine *N*‐acetyltransferase of *Bos taurus* (*btAANAT*), which was used as a reference enzyme. This newly identified enzyme could potentially be a candidate in the currently undefined steps of the melatonin synthesis pathway.

## 2 Materials and Methods

### 2.1 Strains and Culture Media

The strains employed in this study, bacterial or yeast, are listed in Supporting Information S5: Table 1. For plasmid construction and amplification, strain *E. coli* NZYα (NzyTech, Lisboa, Portugal) was used, growing the strain in LB medium (10 g/L tryptone, 5 g/L yeast extract, 5 g/L NaCl) supplemented with 100 µg/L of ampicillin to maintain the plasmid. For protein expression and bioconversion assays, strain *E. coli* Rosetta (DE3) competent cells (Novagen, Darmstadt, Germany) was employed, using as culture medium 2×TY medium (16 g/L tryptone, 10 g/L yeast extract, 5 g/L NaCl) supplemented with 100 µg/L of ampicillin and 34 µg/L chloramphenicol to maintain plasmids. Culture conditions were 37°C and 150 rpm agitation in all cases.

Yeast strains were maintained in YPD medium (20 g/L glucose, 20 g/L peptone, 10 g/L yeast extract). For bioconversion assay, strains carrying plasmids were grown in SC without uracil (20 g/L glucose, 1.7 g/L yeast nitrogen base (YNB) without amino acids and ammonium sulfate (BD Difco, Sparks, MD, USA), 5 g/L ammonium sulfate and 1.9 g/L of SC‐ura drop‐out powder (Formedium, Swaffham, UK)). For solid media, the same medium was supplemented with 16 g/L agar.

### 2.2 Plasmid Construction

The plasmids and primers used in this study are listed in Supporting Information S5: Tables 2 and 3, respectively. Plasmid backbones pAP001 and pGEX‐5X‐1 (GE Healthcare, Chicago, USA) were used for yeast and for bacterial expression, respectively. pAP001 plasmid was constructed using the MoClo‐YTK [34], containing a galactose‐inducible promoter (p*GAL1*), a multiple cloning site with *Bam*HI and *Xho*I sites, and the *ADH1* terminator sequence as expression cassette. The plasmid also contained the selection markers *URA3* and the origin of replication 2micron for *S. cerevisiae*, as well as the selection marker *AmpR* and the origin of replication *OriE* for *E. coli*. Full description of parts and sequence of pAP001 plasmid is contained in Supporting Information S5: Tables 4 and 5.

Genes *IAT4* (YDR391C) and *PAA1* from *S. cerevisiae* were PCR amplified using genomic DNA of yeast strain BY4743. Positive control gene *btAANAT* was amplified from the plasmid pCfB2628 [35]. Phusion DNA polymerase (Thermo Scientific, Waltham, MA, USA) was used for amplification, using primer pairs that introduce a *Bam*HI site and an *Xho*I site to clone in plasmid. The primers pairs were *AANAT* F *Bam*HI/*AANAT* R *Xho*I, *PAA1* F *Bam*HI/*PAA1* R *Xho*I, and *IAT4* F *Bam*HI/*IAT4* R *Xho*I, for *AANAT, PAA1* and *IAT4*, respectively. The resulting PCR fragments were digested with *Bam*HI and *Xho*I and cloned on both plasmids pAP001 and pGEX‐5X‐1. Positive transformants were validated by colony PCR and sequencing using the pair primers check pAP001 F/check pAP001 R and pGEX seq F/pGEX seq R for pAP001 and pGEX‐5X‐1, respectively. For plasmid development used in the *IAT4* overexpression assay, a procedure similar to that described above was followed. The digested fragment of *IAT4* was cloned onto the episomal plasmid p426gpd [36] previously digested with *Bam*HI and *Xho*I. Primers GP2F (*IAT4*) and GV2R (*IAT4*) (Supporting Information S5: Table 3) were used for amplification of *IAT4*, using Phusion U Hot Start polymerase (Thermo Scientific, Waltham, Massachusetts, USA) to subsequent USER cloning on plasmid pCfB2988 [37].

### 2.3 Homology, Molecular Docking, and Phylogenetic Analysis

The Foldseek server [30] were used to search for possible candidate genes with *N*‐acetyltransferase function. The *AANAT* structure of *Homo sapiens* (AF‐Q16613‐F1) and the *psmF* (NCBI Reference Sequence: WP_020929557.1) structure of *Streptomyces griseofuscus* (AF‐W8QGX9‐F1) obtained from the AlphaFold database [31, 38] were chosen as templates. As parameters, a search was performed in Foldseek applying the taxonomy filter to *S. cerevisiae*, 3Di/AA mode, and on the AlphaFold/Proteome v4 database and AlphaFold/UniProt50 v4 database. Molecular docking simulations were performed by the SwissDock 2024 server (https://www.swissdock.ch/) [39, 40], using the AutoDock Vina mode with sampling exhaustivity of 10. The substrate of 5‐metoxytryptamine was introduced as the SMILE code. The windows of the active pockets of the proteins were chosen using information from the crystal‐resolved structure of *Ovis aries AANAT* (1L0C), which was the only one resolved as a crystal structure. The space window was a cube of 10–15 Å around the catalytic position of the *AANAT* binding site. The results were visualised using the SwissDock 2024 server and the conformation between 5‐methoxytryptamine and the proteins with the highest affinity scores was determined. The strength of the ligand‐receptor binding determines the scoring function, so the lower the calculated affinity (kcal/mol), the higher the affinity between the potential enzyme and its substrate.

The phylogenetic tree was constructed using Seaview [41] based on the amino acid sequences of some representative organisms and yeast protein candidates encoded by the genes: *IAT4*, *HPA2*, *PAA1* (from *S. cerevisiae*), *Bos taurus AANAT*, *Ovis aries AANAT*, *Homo sapiens AANAT*, *Oryza sativa SNAT* (1 and 2 type), *Arabidopsis thaliana SNAT*, *Aedes aegypti AANAT*, *Drosophila melanogaster AANAT*, *psmF* (from *Streptomyces griseofuscus)*. Phylogenetic analysis was performed by using Parsimony algorithm. The parameters were “Trees‐Parsimony‐Bootstrap method”. The number of Bootstrap replications was 1000, and ignore all gap sites was employed.

### 2.4 Bioconversion Assays

For *E. coli* bioconversion assay, plasmids pGEX‐5X‐1 with the different genes *IAT4*, *PAA1,* and *btAANAT* were used. These plasmids were transformed into Rosetta (DE3) competent cells (Novagen, Darmstadt, Germany), using empty vector transformation as the negative control. Transformants were grown under 150 rpm shaking at 37°C overnight in 15 mL tubes containing 5 mL of LB medium, supplemented with 100 µg/L of ampicillin and 34 µg/L of chloramphenicol. Precultures were inoculated into 1.5 mL of 2×TY medium, also supplemented with ampicillin and chloramphenicol as described before, to reach an initial OD_600_ of 0.1. When the cultures reached an OD_600_ of 0.6, the expression of the cloned gene was induced by adding to the culture isopropyl‐β‐d‐thiogalactopyranoside (IPTG) to final concentration of 0.25 mM and 5‐methoxytryptamine (substrate) was also added to a final concentration of 1 mM. Cultures were grown during 24 h, at 28°C under 300 rpm orbital shaking. Final samples were taken and stored at −20°C until extraction and HPLC‐FLD analysis.

For *S. cerevisiae* bioconversion assay, a vector‐based inducible overexpression system was employed. To overexpress the genes *IAT4, PAA1,* and *btAANAT*, vector pAP001 was used, a high‐copy number vector which enables strong inducible expression by adding galactose in the culture medium. The final constructed plasmids with genes of interest were transformed into BY4743 yeast strain (EUROSCARF, Oberursel, Germany). Empty vector was also transformed and used as a negative control in the assays. Individual colonies of each transformants were grown in 15 mL tubes containing 5 mL of SC without uracil (SC‐ura) medium at 28°C, overnight and under continuous shaking at 150 rpm. Pre‐inoculum was inoculated into 1.5 mL of fresh SC‐ura medium with 0.1% of glucose to reach an initial OD_600_ of 0.1 in a 24‐well microtiter plate of 2 mL well capacity. Plates were incubated at 28°C under constant shaking in a microplate orbital shaker at 300 rpm. After 2 h of initial inoculation, galactose was added to a final concentration of 2% to induce the expression of the gene of interest and 5‐methoxytryptamine (substrate) was also added to final concentration of 1 mM. Cultures were grown for 48 h and samples were taken and stored at −20°C until extraction and analysis.

Melatonin was determined using a HPLC‐FLD. The supernatant stored in the freezer was combined with methanol at a 50:50 ratio and filtered using nylon syringe filters with a pore size of 0.22 μm and a diameter of 13 mm. Chromatographic analysis was conducted using an AccucoreTM C18 column (4.6 × 150 mm, 2.6 μm; Thermo Fisher Scientific, Waltham, MA, USA) with mobile phases A (0.1% formic acid in water) and B (0.1% formic acid in acetonitrile). The flow rate was set at 0.8 mL/min, and the injection volume was 20 μL. The gradient program was as follows: 0–2 min, 80% A, 20% B; 2–7 min, a gradient from 20% to 44% B; 7–11 min, 10% A, 90% B; 11–17 min, 80% A, 20% B. The column temperature was maintained at 30°C, and samples were kept at 10°C. The excitation and emission wavelengths were set at 286 nm and 350 nm, respectively. A fluorescence detector equipped with an Acquity ARC core (Waters, Milford, MA, USA) (Waters 2575 Fluorescence), a quaternary pump, an autosampler, and a degasser was employed. Calibration curves were generated with melatonin standards.

### 2.5 Expression and Purification of Proteins

For the expression and purification procedure, we used the protocol described by Harper and Speicher [42], with some modifications. The GST fusion protein generated after cloning the coding sequence into the pGEX‐5X‐1 plasmid was transformed into *E. coli* Rosetta (DE3) competent cells (Novagen, Darmstadt, Germany) strain. An overnight preculture of the strain in 10 mL LB medium containing ampicillin (100 µg/L) and chloramphenicol (34 µg/L) was inoculated into 400 mL of 2×TY medium with the same antibiotic concentrations. The culture was incubated at 37°C for 4 h until it reached an OD_600_ of 0.5–0.7. Then, 1 mM of IPTG was added and the culture was incubated at 28°C and 150 rpm orbital shaking for 16 h. The purification procedure was made using Glutathione Sepharose 4B resin (GE Healthcare Life Sciences), and purification was performed according to the manufacturer's recommendations. For concentration and buffer exchange, Amicon Ultra‐0.5 mL Centrifugal Filters 30 K (Merck KGaA, Darmstadt, Germany) were used. Protein concentration was determined using direct measurements of absorbance at 280 nm in a NanoDropTM 2000 microvolume UV‐Vis Spectrophotometer (Thermo Fisher Scientific). To analyse the quality of protein purification, SDS‐PAGE using 12% polyacrylamide gels, followed by Coomassie blue staining was employed.

### 2.6 Iat4 Enzyme Kinetics Measurements

Iat4 protein activity was measured by DTNB‐based quantification as described previously [43]. First, a basic characterisation was carried out to determine the optimal conditions for the enzyme assays in two key environments, pH and temperature, using serotonin as substrate. To evaluate enzyme activity at different temperatures and pHs, 0.1 µg of purified recombinant Iat4 protein was incubated in a total volume of 100 µL containing 0.5 mM serotonin and 0.2 mM acetyl‐CoA in 10 mM Tris‐EDTA (TE) buffer. For the temperature test, the pH was set at 8.8 and for the pH test, the temperature was set at 37°C. After 15 min of reaction, an equal volume of quenching buffer containing DTNB was added to stop the reaction. Samples were transferred to a microplate before analysis by the spectrophotometer at λ = 412 nm. For substrate affinity (*K*_m_) and the maximum reaction rate (*V*max) calculation, several concentrations of each indolamine compound (serotonin, tryptamine and 5‐metoxytriptamine) were tested, incubating the reaction a 37°C in pH 8.8 during 15 min.

### 2.7 Overexpression of *IAT4* in a Serotonin‐Overproducing Strain

To test the functionality of the *IAT4* gene in vivo, this gene was overexpressed in a serotonin overproducing strain. The construction of this strain was described by Planells‐Cárcel et al. [44] and it was as follows: the sequences of the TDC gene from *Clostridium sporogenes* (*Cs*TDC) and T5H from *Oryza sativa* (*Os*T5H) were chemically synthesised by Twist Bioscience (California, USA) and optimised for the use of codons from *S. cerevisiae*. These genes *Cs*TDC and *Os*T5H were cloned in the multicopy integration plasmid pCfB2803 [37]. For this purpose, both genes and bidirectional promoter TEF1p‐PGK1p were amplified by PCR from synthetic genes and plasmid pCfB2628 [37] respectively, using Phusion U Hot Start polymerase (Thermo Scientific, Waltham, Massachusetts, USA) and specific primers for USER cloning CsTDC‐GV1R, CsTDC‐GP1F, OsT5H‐GV2R, OsT5H‐GP2F, PG1R(TEF1p), and PG2R(PGK1p) (Supporting Information S5: Table 3). The feedback inhibition‐insensitive ARO4* gene was cloned in plasmid pCfB2797 HIS3, a derivate plasmid from plasmid pCfB2797 [37] changing the selection marker from *URA3* to *HIS3*. To do so, primers PV2F (GPDp) and GV2R (*ARO4*), were used. For successful cloning, several *E. coli* colonies were subjected to PCR with ADH1_test F and CYC1_test R primers (Supporting Information S5: Table 3), followed by Sanger sequencing (Eurofins genomics, Ebersberg, Germany) for positive colonies' verification. Resulting integrator vectors were linearised by FastDigest *Not*I (Thermo scientific, Waltham, Massachusetts, USA) and transformed into yeast by using PEG/LiAc method [45]. To generate a control strain and to remove the remaining auxotrophies from engineered strains, each strain was transformed with its respective linear product derived from the corresponding empty multiple integration plasmid (pCfB2803 for *LEU2*, pCfB2797 HIS3, for *HIS3*). This serotonin overproducing strain was named BS2.

The BS2 strain was transformed in two different ways: one by overexpressing the gene using a high copy number episomal vector (p426gpd *IAT4*); and the other by integrating multiple copies of the gene expression cassette (linear product obtained from pCfB2899 *IAT4*) into the repetitive TY elements. In both cases, the gene was expressed under the control of the strong promoter GPDp. These strains were inoculated at OD_600_ of 0.2 and grown in SC medium (SC‐ura in the strains with episomal vector) for 48 h. The supernatant was combined with methanol to achieve a final 50:50 ratio and then filtered using nylon syringe filters with a pore size of 0.22 μm and a diameter of 13 mm. *N*‐acetylserotonin was determined by using a UHPLC coupled with fluorescence detector (Waters 2575 Fluorescence), using an AccucoreTM C18 column (4.6 × 150 mm, 2.6 μm; Thermo Fisher Scientific, Waltham, MA, USA) with mobile phases A (0.1% formic acid in water) and B (0.1% formic acid in acetonitrile). The flow rate was set at 0.8 mL/min, and the injection volume was 10 μL. The gradient program was as follows: 0–2 min, 90% A, 10% B; 2–7.5 min, a gradient from 10% to 15% B; 7.5–11 min, 10% A, 90% B; 11–17 min, 90% A, 10% B. The column temperature was maintained at 30°C, and samples were kept at 10°C. The excitation and emission wavelengths were set at 286 nm and 340 nm, respectively. For quantification of concentration, calibration curve was generated with analytical standards of *N*‐acetylserotonin obtained from Sigma‐Aldrich (St. Louis, MO, USA).

### 2.8 Statistical Analysis

Experimental results were analysed and compared by statistical ANOVA one‐way analysis using GraphPad Prism (GraphPad Software Inc., San Diego, CA, USA) software (v7.00). Comparisons were corrected by Tukey test and significance was shown in graphs as *p* < 0.05 (*), *p* < 0.01 (**), *p* < 0.001 (***), *p* < 0.0001 (****). All the experiments of bioconversion and *N*‐acetylserotonin production were carried out in triplicates. For the calculation of the kinetic parameters, SigmaPlot Version 15 (Systat Software Inc., San Jose, California) was used to fit the data to a Michaelis–Menten kinetic model. For the goodness of fit of the model, *R* ^2^ greater than 0.9 was considered. *K_m_* and *V*max values were represented as mean and standard deviation obtained according to the model. All assays of kinetics were performed in triplicate.

## 3 Results

### 3.1 The Search for New Indolamine *N*‐Acetyl Transferases in *S. cerevisiae*

To identify genes involved in the *N*‐acetylation of tryptophan‐derived compounds relevant for melatonin synthesis, a search was performed in the Foldseek server. Both servers represent a network‐based tool designed for comparative analysis of protein structures in three‐dimensional space [29, 30]. The main idea behind the use of these servers is the comparison of structures to reveal remarkable similarities of biological importance that may not be discernible by traditional sequence‐based comparisons, since using sequence homology may miss proteins with highly different sequences, but with a similar structure that are able to display the same enzymatic activity.

To cover a larger space of structural diversity but with the certainty of the desired enzymatic reaction, we used as a template the protein structure of the protein encoded by *AANAT* from *Homo sapiens* (*hsAANAT*), a well‐characterized protein of 207 aa representing the mammalian enzyme, and the protein encoded by *psmF* from *Streptomyces griseofuscus*, a bacterial protein of 191 aa that has been demonstrated to have *N*‐acetyltransferase activity previously [46], and it is phylogenetically more distantly related to the mammalian protein. Both structures were analysed using Foldseek [30].

**Figure 2:**
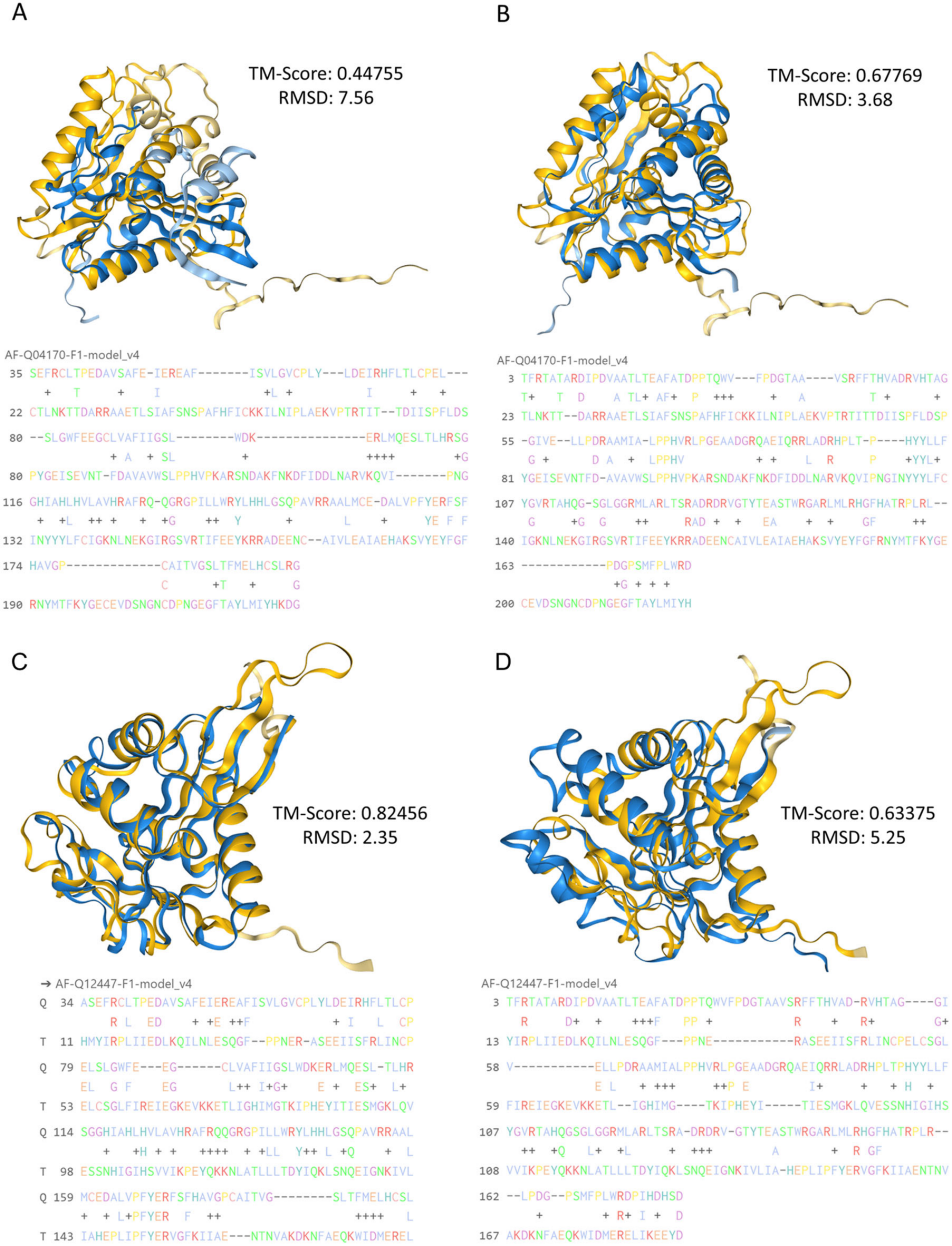
Graphical representation of the structural alignment of Iat4 and Paa1 with Foldseek. (A) Structural alignment of *hsAANAT* (AF‐Q16613‐F1) in blue with Iat4 (AF‐Q04170‐F1) in yellow. (B) Structural alignment of *psmF* (AF‐W8QGX9‐F1) in blue with Iat4 in yellow. (C) Structural alignment of *hs*Aanat (AF‐Q16613‐F1) in blue with Paa1 (AF‐Q12447‐F1) in yellow. (D) Structural alignment of *psmF* (AF‐W8QGX9‐F1) in blue with Paa1 in yellow. Template Modeling Score (TM‐score) quantifies overall structural similarity, with values ranging from 0 (low similarity) to 1 (high similarity). Root Mean Square Deviation (RMSD) measures atomic‐level deviations between two structures, with lower values indicating better alignment and higher values suggesting greater differences.

Supporting Information S6: Table 6 shows the list of proteins with a top score obtained by Foldseek. A taxonomic filter was applied to show only *S. cerevisiae* proteins from the AlphaFold database that have a high similarity to the target proteins used, *hsAANAT* and *psmF*. In the case of *hsAANAT* as a search query, the first ranked candidate is the protein encoded by *PAA1* gene, showing a probable structural similarity from amino acids 34 to 195, with a sequence identity of 21.1%, resulting in a very significant E‐value (4.59·10^−13^). In contrast, in the case of *psmF*, the best match found in the top‐ranked position in the table is the protein encoded by “Uncharacterized protein YDR391C”, named as *IAT4* gene in this study. *IAT4* encodes for a protein of 232 aa in size and has not been characterised to date. In this case, it covers a larger position, being almost the entire protein (from amino acids 3 to 175) and an identity of 17.6%, generating an E‐value of 2.16·10^−9^. Figure 2A,B shows the similarity of *IAT4* with *hsAANAT* and *psmF*. We can observe that the Template Modeling score (TM‐score) in the case of *AANAT* is lower than with the *psmF* protein, being 0.4475 and 0.6777, respectively. In both cases it is close to the TM‐score value of 0.5 that is used to suggest homology, but in the case of comparison with the structure of *psmF* the value is above, highlighting its greater similarity with this gene. In the case of *PAA1*, we can appreciate that the TM‐score against hs*AANAT* is very high, 0.8246, compared to the value obtained against *psmF*, which is 0.6637 (Figure 2C,D). *PAA1* has been previously studied as a possible *N*‐acetyltransferase involved in the melatonin pathway, but there are currently no specific publications linked to *IAT4*. We therefore proceeded with its functional evaluation in vivo and propose to name the gene *IAT4* according to its activity, in reference to “Indolamine AcetylTransferase”.

To complement this protein structure‐based search, a molecular docking simulation was used to assess the affinity of Iat4 for its putative substrate, 5‐methoxytryptamine. As control, the crystal resolved structure of *Ovis aries AANAT* (1L0C) was used to aid the search for the catalytic pocket. The structures generated and a table of calculated affinities for each of the proposed docking models after docking with 5‐methoxytryptamine (5‐MT) are shown in Figure S1. In the case of *AANAT* (Supplementary Figure S1A), we found that the highest affinity of the 5‐MT molecule gives −6.357 kcal/mol. In addition, the position of the molecule is correctly in the expected location found in the crystal resolved structure, although the orientation differs slightly from the crystallographic model due to the absence of the other co‐substrate acetyl‐CoA. In the case of *IAT4* (Figure S1B), it was observed that the calculated affinity is slightly higher, at −6.189 kcal/mol, which would indicate a lower affinity compared to *AANAT*, but with a high similarity between them.

### 3.2 Detection of NAT Activity of *IAT4* by Overexpression in *E. coli* and *S. cerevisiae*

**Figure 3:**
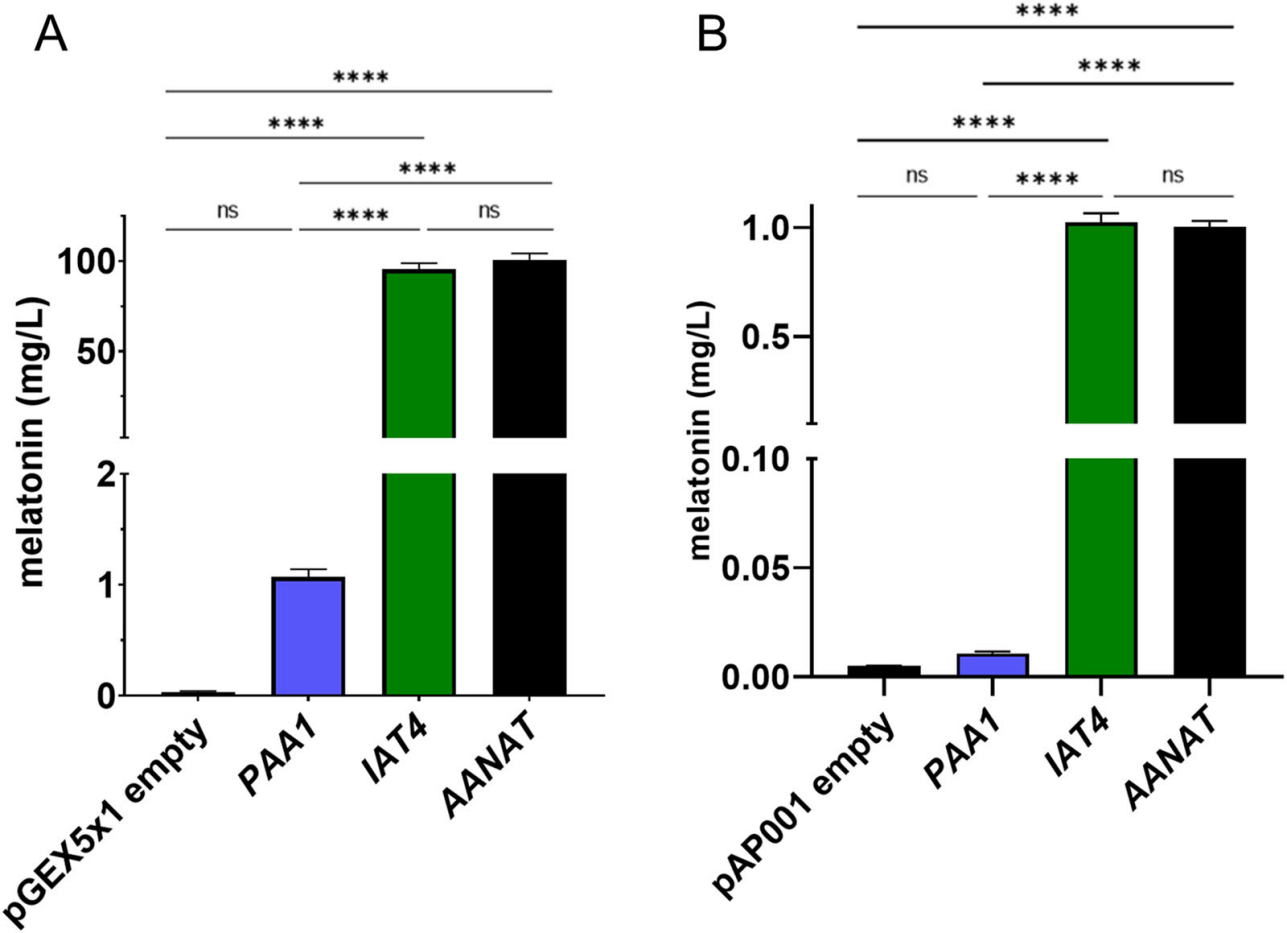
Bioconversion assays of 5‐metoxytryptamine by *PAA1*, *IAT4,* and *btAANAT* enzymes in different protein expression systems. (A) Bacterial expression system using the pGEX‐5X‐1 vector transformed into *E. coli* Rossetta cells. (B) Yeast expression system using the pAP001 vector transformed into *S. cerevisiae* strain BY4743. Values are expressed as means ± SD and each assay was performed in triplicate. One‐way ANOVA was used to determine statistical significance between the mean values for melatonin production of the control, *PAA1*, *IAT4,* and *btAANAT* overexpressing strains (ns: not significant; **** = *p* < 0.0001).

To evaluate the *N*‐acetyltransferase activity of Iat4 enzyme, several bioconversion experiments were performed. The *IAT4* gene was overexpressed in an inducible expression system for both *E. coli* and *S. cerevisiae*, similar to that described by Bisquert et al. [27]. The idea was to observe in both biological systems whether the strains, after induction of protein expression, showed a conversion of the supplemented 5‐methoxytryptamine to melatonin. Both *PAA1* and *btAANAT* were overexpressed in the same systems to compare the bioconversion capacity of both against *IAT4*, and a strain with an empty plasmid was also used as a negative control. Figure 3A shows the melatonin production by each of the transformed *E. coli* strains after the addition of 5‐methoxytryptamine to the culture. It can be noted that the melatonin production of the Iat4 enzyme reaches levels of 95 mg/L, being very similar to the values obtained in the positive control (*btAANAT* ≈ 100 mg/L). In contrast, we can observe that the overexpression of the *PAA1* gene, even though it showed melatonin bioconversion, reached around 1 mg/L, which was a significantly lower production than that obtained with *IAT4* gene. In the case of overexpression of these genes in *S. cerevisiae* system (Figure 3B), the results show a similar pattern, reaching values of 1 mg/L for both *IAT4* and *btAANAT* overexpression. In contrast, *PAA1* gene overexpression shows nonsignificant amounts with respect to the negative control. As already observed in our previous study [27], it is surprising that the highest yield of acetylated product is obtained in a heterologous organism such as *E. coli* than in its own host, *S. cerevisiae*. Our explanation for these differences in the enzymatic conversion rate in the two organisms is a consequence of the higher overexpression levels of recombinant proteins in *E. coli* than in *S. cerevisiae*. It is well known that *E. coli* has a faster growth rate than *S. cerevisiae* and that plasmids are typically small and replicate rapidly. All of these characteristics result in high levels of protein expression in *E. coli* and, consequently, higher conversion rates by the overexpressed enzyme. However, other factors, such as strong overexpression being harmful to yeast growth or active repression of protein production, may also contribute to the low expression levels observed in *S. cerevisiae* [47]. Additionally, differences in substrate uptake, cofactor availability, and feedback inhibition mechanisms may influence conversion rates and productivity. Morcillo‐Parra et al. [22, 23] proposed that melatonin could act as a signalling molecule in yeast under stress conditions and that its biosynthetic pathways should therefore be highly regulated at different levels.

### 3.3 Expression and Characterisation of the Purified Enzyme Encoded by Iat4

Since the *N*‐acetyltransferase activity of Iat4 was evident in the bioconversion of 5‐methoxytryptamine to melatonin, we proceeded with its in vitro enzymatic characterization, aiming to obtain its kinetic parameters and determine its specificity for various indolamine substrates. For this purpose, Iat4 was cloned as a fusion protein with GST using the plasmid pGEX‐5X‐1, which allows posterior purification. The purity of the enzyme was higher than 95% and showed the correct size of 52.81 kDa of the Iat4 protein together with the GST tag (Figure S2).

**Figure 4:**
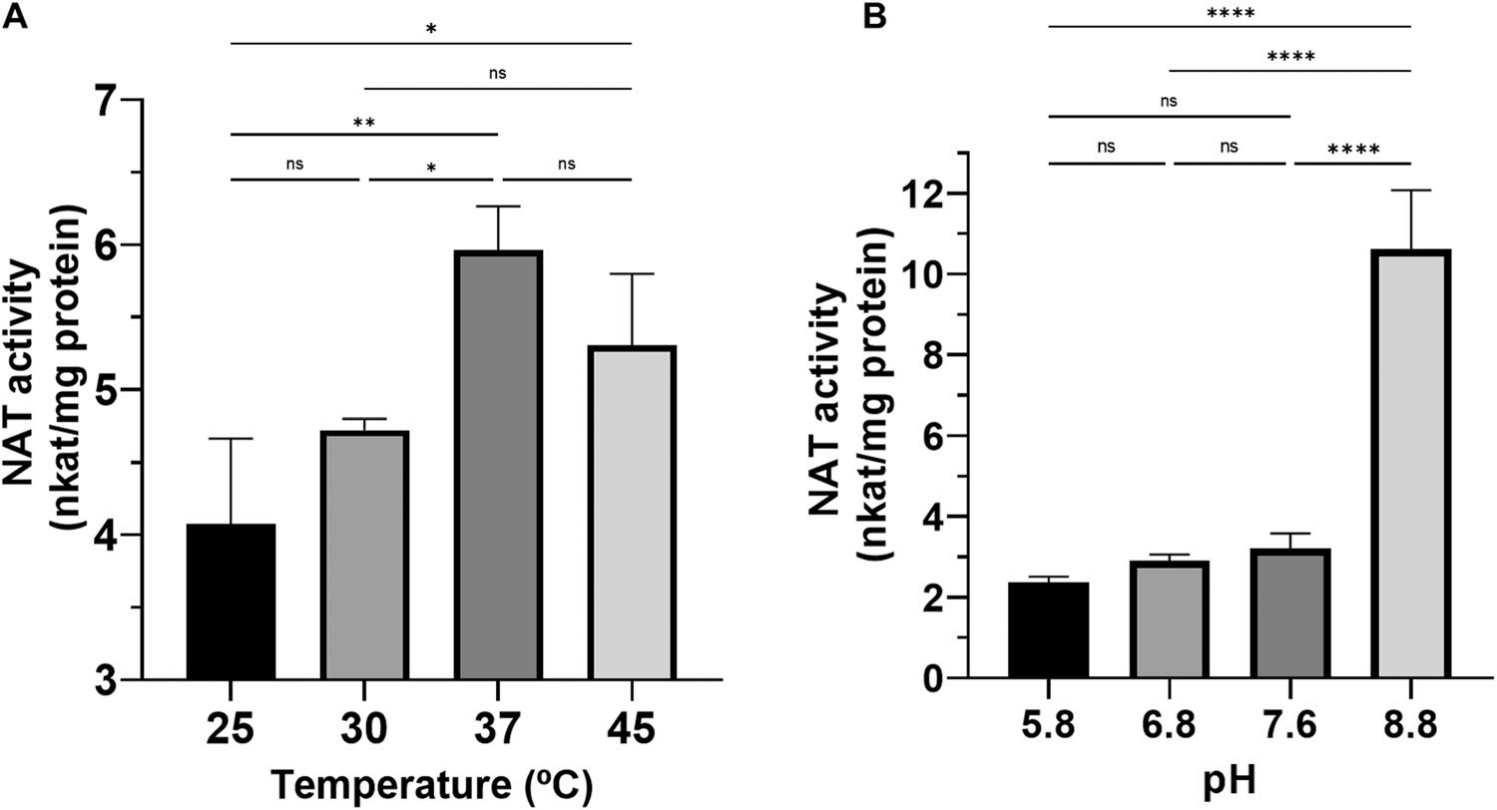
Enzyme activity of *Iat4* protein as a function of temperature (A) or pH (B). For enzymatic assay, serotonin was used as substrate and the same amount of protein was used in all assays (1 mg). Each assay was performed in triplicate and data are expressed as mean ± SD. One‐way ANOVA followed by Tukey's multiple comparison test analysis was used to compare NAT activity between different temperature or pH conditions (ns: not significant; *= *p* < 0.05; **= *p* < 0.01; ****= *p* < 0.0001).

**Figure 5:**
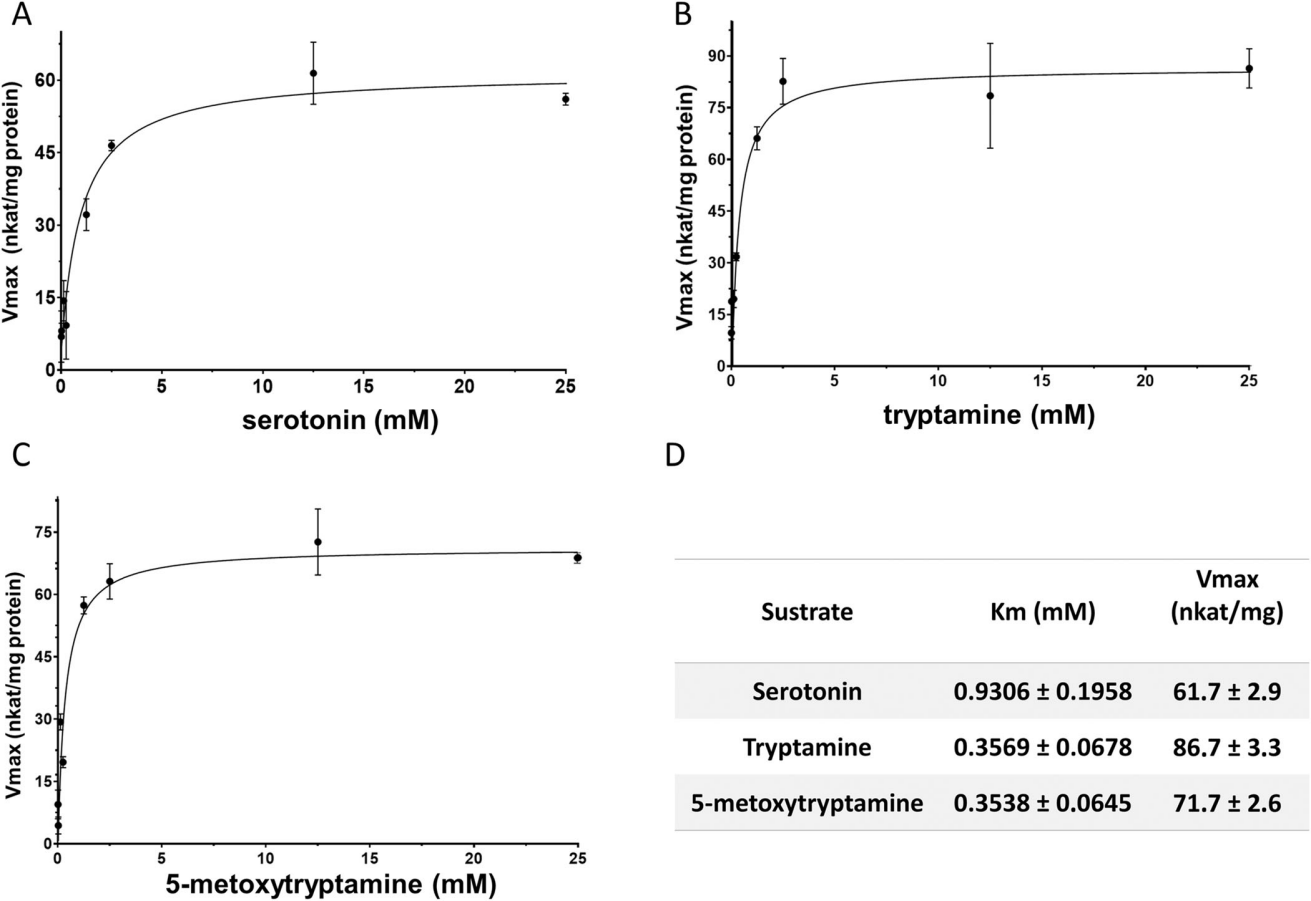
Michaelis–Menten enzyme kinetics for the Iat4 enzyme using indolamines as substrate: (A) Serotonin, (B) Tryptamine, (C) 5‐methoxytryptamine. (D) Table with the values of the affinity constant (*K_m_*) and the maximum velocity (*V*max). Purified Iat4 (1 mg) was incubated with different concentrations of indolamines and acetyl‐CoA (0.2 mM) for 15 min at 37°C and pH 8.8. Values were fitted to Michaelis–Menten model, using *R* ^2^ as goodness of fit (serotonin: *R*² = 0.924; tryptamine: *R*² = 0.928; 5‐metoxytryptamine: *R*² = 0.937). All kinetics were performed with 7 points performed in triplicates. Values are expressed as mean ± SD, *n* = 3.

Before the calculation of kinetic parameters, we performed activity tests using serotonin as a substrate at different pH and temperature to obtain the optimal parameters for the enzyme assays. The highest enzyme activity was found near 37°C and at pH 8.8 (Figure 4). Substrate specificity for Iat4 was studied using several indolamines involved in the melatonin production pathway such as tryptamine, serotonin and 5‐metoxytryptamine. Using an enzymatic assay with DTNB‐based quantification [43], kinetic curves were plotted for each substrate (Figure 5A–C), as well as their kinetic parameters (Figure 5D). In Figure 5D we can see the *K*_*m*_ and maximum velocity (*V*max) values of Iat4 for each indolamine substrate tested. As can be seen, the affinity for 5‐methoxytryptamine and tryptamine was similar, with a *K*_*m*_ of 0.35 mM for both substrates, but lower than the *K*_*m*_ calculated in the case of serotonin (0.93 mM).

To compare the kinetic parameters obtained for Iat4, we also determined the *K_m_* and *V*max of the previously proposed indolamine *N*‐acetyltransferases Paa1 [25] and Hpa2 [27] by the same enzymatic assay (Figure S3). Paa1 showed a *K_m_* value of 4.25 ± 1.46 mM, which is similar to that obtained by Ganguly et al. [25] (5.1 mM for serotonin) and more than four times higher than that shown by Iat4 (0.93 mM). Similarly, a *K_m_* value of 6.82 ± 1.62 was obtained for Hpa2, which showed an even lower affinity to serotonin than Paa1.

### 3.4 Overexpression of the *IAT4* Gene in a Serotonin‐Overproducing Strain

**Figure 6:**
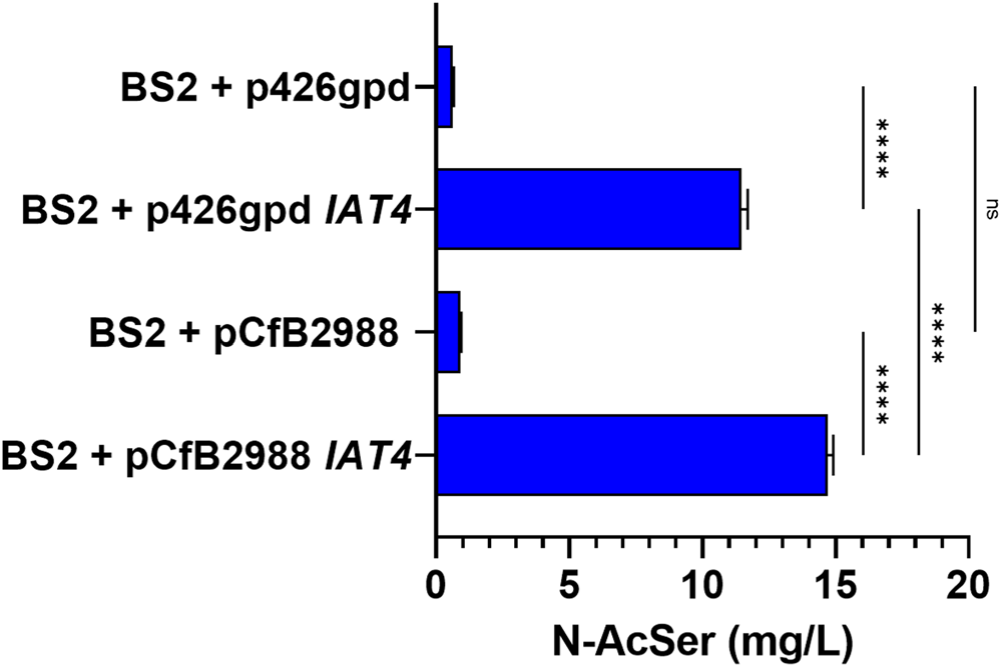
Production of *N*‐acetylserotonin (*N*‐AcSer) by expression of the *IAT4* gene in a serotonin‐overproducing strain. The strains used are the serotonin overproducing strain (BS2) with different variations: the BS2 strain with *IAT4* expression on an episomal plasmid (BS2 + p426gpd *IAT4*), the control strain transformed with empty plasmid (BS2 + p426gpd), the BS2 strain with integration of multiple copies of *IAT4* (BS2 + pCfB2988 *IAT4*), and the control strain with integration of empty backbone pCfB2899 (BS2 + pCfB2988). Values are expressed as mean ± SD. One‐way ANOVA and Tukey's multiple comparison test were used to compare the *N*‐acetylserotonin produced between the different strains (ns: not significant; ****= *p* < 0.0001).

To gain further insight into the role of *IAT4* in *N*‐acetylserotonin biosynthesis in vivo, we overexpressed this gene in a serotonin‐overproducer strain, constructed previously in our lab [44], named BS2. While plasmid‐based gene expression often surpasses that of genomic integration, the latter occurs in every cell of the yeast population and enhances genetic stability [34, 48]. For this reason, we evaluated *N*‐acetylserotonin production after the *IAT4* overexpression using both methods: episomal plasmid overexpression and multiple‐copy genomic integration using p426gpd or pCfB2988 vectors, respectively. Therefore, two strains with different expression of *IAT4* were created: strain BS2 + p426gpd *IAT4* was developed through plasmid‐based expression using p426gpd *IAT4*, while strain BS2 + pCfB2988 *IAT4* was created by multiple integration of the expression cassette GPDp‐*IAT4* obtained by linearization of pCfB2988 *IAT4* previously. In addition, BS2 was transformed with empty vectors from each system to generate control strains BS2 + p426gpd and BS2 + pCfB2988. All the strains were grown in SD medium and the final production of *N*‐acetylserotonin was analysed after 72 h. As it can be seen in Figure 6, *N*‐acetylserotonin production was much higher in the strains overexpressing *IAT4*. In the case of BS2 + p426gpd *IAT4*, 11.3 mg/L *N*‐acetylserotonin was obtained, compared to 0.6 mg/L in the control strain BS2 + p426gpd, representing ~18.8‐fold improvement on *N*‐acetyl serotonin production. In the case of the strain BS2 + pCfB2988 *IAT4*, we observed a production of 14.5 mg/L compared to 0.9 mg/L for the respective control strain BS2 + pCfB2988. This represents an approximately 16‐fold increase in *N*‐acetyl serotonin production.

## 4 Discussion

Melatonin synthesis by yeasts has been reported on several occasions, mostly in a fermentative context [10, 18, 19, 20, 49]. However, the genetic determinants involved in its synthesis remain undefined. *PAA1* is the only gene described in the literature that is involved in the melatonin synthesis pathway [25], which was found based on its sequence homology with human *AANAT*. However, subsequent studies showed that its function in vivo is focused on the *N*‐acetylation of polyamines such as putrescine, spermidine, and spermine [26]. In addition, in our previous study [27], we described the existence of other enzymes that might be involved in the *N*‐acetylation of serotonin such as *HPA2*. For this reason, in this study we focused on the search for *N*‐acetyltransferase enzymes that could be involved in melatonin synthesis.

*AANAT* or *SNAT* is a universal enzyme that is present in a multitude of organisms, beyond vertebrates, where it was first described. In recent years, several SNATs have been identified and characterised in plants, bacteria, and archaea [50, 51, 52]. The *SNAT* enzyme belongs to the GCN5‐related *N*‐acetyltransferase (GNAT) superfamily, a family of enzymes that are capable of acetylating a wide variety of substrates, from small molecules to the N‐terminal domain of proteins [53]. All enzymes in this family exhibit highly conserved structure, known as GNAT fold, which typically consists of 6–7 anti‐parallel β‐strands and four α‐helices arranged in a specific topology. Despite sharing the same fold, the GNAT superfamily is very diverse in terms of amino acid sequence and substrate usage. Furthermore, sequence similarity is sometimes low even when the substrates are similar and even minor changes in the GNAT fold can lead to changes in substrate specificity, highlighting the diversity and adaptability of these enzymes [54, 55]. This suggests that sequence homology analysis may not be the best option for finding genes homologous to SNAT. Advances in computation and the emergence of the AlphaFold structural prediction database [38] have enabled the field of homolog search to grow [56]. In addition, the relationship between enzymes and their substrate interactions can be studied more easily and quickly using molecular docking simulations. These new approaches have allowed to find and assign functions to still uncharacterised genes as well as to discover new metabolic pathways in other organisms [29].

In this study, by using bioinformatics tools such as Foldseek and employing a newly discovered bacterial *AANAT* enzyme, *psmF*, we have been able to identify and determine the function of the *IAT4* gene. *psmF* is part of an enzymatic complex of *Streptomyces griseofuscus* involved in the biosynthesis of physostigmine, a parasympathomimetic drug [46]. During the synthesis of this compound there is an intermediate step where *N*‐acetylation of serotonin to *N*‐acetylserotonin takes place. The fact that *psmF* has a structure with recognised *N*‐acetyltransferase activity and is phylogenetically distant from the mammalian *AANAT* gene (Figure S4), showing a structural difference between them, has allowed us to use *psmF* as a template to find a new functional gene in yeast that has *N*‐acetyltransferase activity and may be involved in the melatonin synthesis pathway in *S. cerevisiae*. In terms of computational prediction, the result of the docking simulation also indicated a high affinity of *IAT4* for its substrate 5‐methoxytryptamine, with values similar to those obtained for mammalian *AANAT*, confirming the possible involvement of this gene in the synthesis of melatonin.

In vivo bioconversion assays showed a good capacity of *IAT4* to acetylate 5‐methoxytryptamine. These assays are good approaches to screen and evaluate potential enzyme candidates, being commonly tested in the *E. coli* system due to higher expression levels of recombinant proteins compared to *S. cerevisiae* [27]. In the case of *IAT4*, we observed high levels of conversion in both organisms, something that does not occur in *PAA1*, suggesting that the function of *IAT4* may be more specific for these substrates than in the case of *PAA1* or of *HPA2*, a new arylalkylamine *N*‐acetyltransferase recently described by us [27].

Kinetic parameters measured for purified Iat4 showed low *K*_*m*_ for indolamine compounds involved in the melatonin synthesis pathway (Figure 5D), which means that the enzyme could achieve significant reaction rates even at low substrate concentrations. Interestingly, the affinity for serotonin is slightly lower than for 5‐methoxytryptamine, which may indicate that the melatonin synthesis pathway may predominate for this flux as opposed to the synthesis pathway via acetylation of serotonin to *N*‐acetylserotonin. This alternative pathway has previously been proposed in other organisms by other authors [1, 57]. Tan et al. [1] hypothesised that this alternative pathway is predominant in some plants, bacteria, and perhaps yeast, and may also occur in animals. These authors also suggested that these reactions in other organisms may not generally be catalysed by enzymes identical to those studied in vertebrate pineal glands, as we have shown in this study. However, both pathways have been found to be present in yeast, so it could be a combination of both pathways [24]. In the literature, mammalian Aanat *K*_*m*_ measurements are shown to be in the order of 1.3 mM for serotonin and 0.13 mM for tryptamine [58], similar values to those obtained for Iat4, in agreement with those reported in the bioconversion assays. In the case of Paa1, Ganguly et al. [25] reported a *K*_*m*_ of 2.7 mM for 5‐methoxytryptamine, 5.1 mM for serotonin and 4.7 mM for tryptamine. These values are more than five times higher than those shown by Iat4 for 5‐methoxytryptamine. Furthermore, overexpression of *IAT4* in the serotonin‐overproducing strain shows a considerable increase in *N*‐acetylserotonin. All these evidence pointed out that *IAT4* encodes an enzyme with high affinity for different indolamines and shows a higher acetylation activity than *PAA1* and other possible genes with *N*‐acetyltransferase activity in *S. cerevisiae*.

To date, the only clear indication in the literature about this gene of unknown function is that its deletion confers sensitivity to metal deficiency [59] and, as consequence, these authors proposed its involvement in zinc homeostasis. At the transcriptional level, it is dependent on the transcription factor *BDF1*, which is involved in the salt stress response [60], and it has been reported for its induction by salt and by oxidative stress in a massive transcriptional study in the presence of hydrogen peroxide and menadione [61]. This significant induction of this gene in the presence of powerful oxidants can be connected with our proposal that the primary role of melatonin synthesis is to protect yeast cells against oxidative stress and UV radiation [15]. In any case, future experimental work should be done to demonstrate that the main role of this enzyme is the synthesis of melatonin. The canonical experiment to prove the involvement of a specific gene in a biosynthetic pathway is the comparison between the wild type and its mutant in terms of substrate conversion or yield production. However, in our experience, this experiment is not always conclusive in the case of melatonin synthesis because the native production of this molecule is very low, close to the limit of detection, and the presence of other acetyltransferases, with the capacity to convert indolamines, makes it very difficult to detect differences in substrate conversion between wild‐type and mutant strains [27].

In summary, we have demonstrated that the availability of new bioinformatic tools, most of them based on artificial intelligence (AI), can definitively contribute to the prediction of the function of many proteins of unknown function. Following this strategy, we found a protein with the highest *N*‐acetyltransferase (NAT) specificity for different types of indolamines described so far in *S. cerevisiae*, which we propose as the main indolamine NAT in this organism. To the best of our knowledge, we consider this result to be highly relevant because a better understanding of the biosynthetic pathways involved in the synthesis of bioactive molecules will allow the selection of yeast strains with enhanced properties for the production of human health‐promoting compounds. It is also noteworthy that our studies of enzymatic kinetics supported the idea of an alternative pathway in *S. cerevisiae* in which serotonin is first *O*‐methylated to 5‐methoxytryptamine (5‐MT) and then 5‐MT is *N*‐acetylated to melatonin. However, it remains to be seen whether the enzymes involved in melatonin synthesis are exclusive to this process or, as already mentioned by Ganguly et al. [25], the synthesis of this molecule may reflect the opportunistic action of otherwise unrelated metabolic enzymes with broad functions (aromatic hydroxylation, decarboxylation, *N*‐acetylation, *O*‐methylation). An important consequence of arylalkylamine acetylation is that it would prevent the conversion of an amine to an aldehyde, which is potentially toxic due to high reactivity and non‐selectivity. Arylalkylamines are present in the environment and can also be generated in cells by the decarboxylation of aromatic amino acids, for example, tryptamine from tryptophan. Therefore, it seems reasonable to assume that the primary function of *AANAT* early in evolution was detoxification [25]. Finally, we also consider the discovery of this new enzyme as a hallmark for the development of yeast cell factories that produce interesting indolamines, such as serotonin, *N*‐acetylserotonin, and melatonin [43]. The activity and selectivity of these new enzymes can be further improved using artificial intelligence and machine learning tools within a few iterations of the design‐build‐test‐learn cycle [62].

## Author Contributions

**Andrés Planells‐Cárcel:** conceptualization, methodology, formal analysis, visualization, validation, investigation, and writing – original draft. **Sandra Sanchez‐Mart**i: methodology, validation, and investigation. **Sara Muñiz‐Calvo:** conceptualization, methodology, investigation, supervision writing – review and editing, methodology, and validation. **José Manuel Guillamon:** funding acquisition, resources, project administration, supervision, writing – review and editing.

## Conflicts of Interest

The authors declare no conflicts of interest.

## Supporting information


**Figurementary Figure 1**. Molecular docking analysis of the binding capacity of 5‐metoxytryptamine (5‐MT) with *Iat4* and *oaAANAT*. (A) Representation of the model of 5‐MT binding to *oaAANAT,* together with the different affinity values of the predicted models. (B) Representation of the model of 5‐MT binding to *Iat4,* together with the different affinity values of the predicted models.

**Figurementary Figure 2**. SDS‐PAGE of *Iat4* expression and purification. (a) Non‐induced cells, (b) IPTG‐induced cells, (c) Cell lysis, (d) Soluble fraction, (e) Flow through, (f) Wash, (g) Purified extract.

**Figurementary Figure 3**. Michaelis–Menten enzyme kinetics for the enzymes. (A) *IAT4* (B) *PAA1,* and (C) *HPA2*, using serotonin as substrate. (D) Table with the values of the affinity constant (*K_m_*) and the maximum velocity (*V*max). Values are expressed as mean ± SD. All kinetics were performed with 7 points performed in triplicate. Values were fitted to Michaelis–Menten model, using *R* ^2^ as goodness of fit (*PAA1*: *R*² = 0.825; *HPA2*: *R*² = 0.939; *IAT4*: *R*² = 0.924).

**Figurementary Figure 4**. Phylogenetic tree of *N*‐acetyltransferases (NAT). The phylogenetic tree was constructed using Seaview based on the amino acid sequences of some representative organisms and yeast proteins candidates, being represented by their gene name: *IAT4, HPA2, PAA1* (from *S. cerevisiae*), *Bos taurus btAANAT, Ovis aries oaAANAT, Homo sapiens hsAANAT, Oryza sativa osSNAT* (Version 1 and 2), *Arabidopsis thaliana SNAT, Aedes aegypti AANAT, Drosophila melanogaster AANAT, psmF* (from *Streptomyces griseofuscus*). Phylogenetic analysis was performed by using the Parsimony algorithm. The parameters were “Trees‐Parsimony‐Bootstrap method”. The number of Bootstrap replications was 1000, and ignore all gap sites was employed.

Supplementary_Tables.

**Supplementary Table 6**. *S. cerevisiae* proteins with homology similar to *hsAANAT* and *psmF*. Database used by FoldSeek was AlphaFold/Proteome database v4, choosing *S. cerevisiae* as taxonomic filter. Results are ordered according to their score, based on its statistical significance. *IAT4* and *PAA1* protein in each of the analyses are highlighted in orange.

## Data Availability

The data that support the findings of this study are available from the corresponding author upon reasonable request.
