# Supplementary figures and images for: IAT4, a New Indolamine N‐Acetyltransferase in Saccharomyces cerevisiae Involved in Melatonin Biosynthesis
Source: J Pineal Res. 2025 May 9;77(3):e70053. doi: 10.1111/jpi.70053 (PMC12063518; doi:10.1111/jpi.70053)

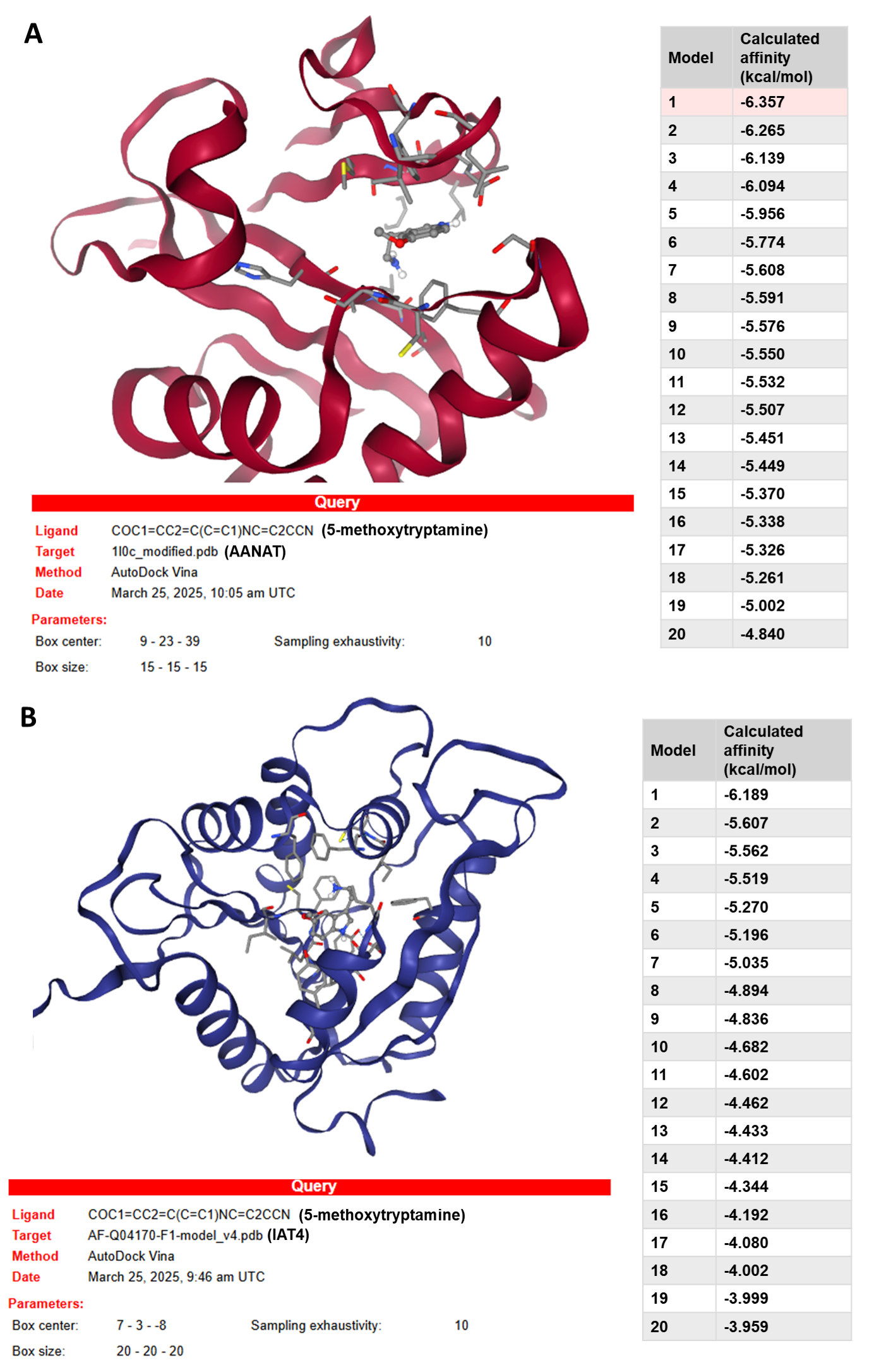

Supplement: Supplementary file 1 — Figurementary Figure 1. Molecular docking analysis of the binding capacity of 5‐metoxytryptamine (5‐MT) with Iat4 and oaAANAT. (A) Representation of the model of 5‐MT binding to oaAANAT, together with the different affinity values of the predicted models. (B) Representation of the model of 5‐MT binding to Iat4, together with the different affinity values of the predicted models. [file JPI-77-e70053-s005.png]

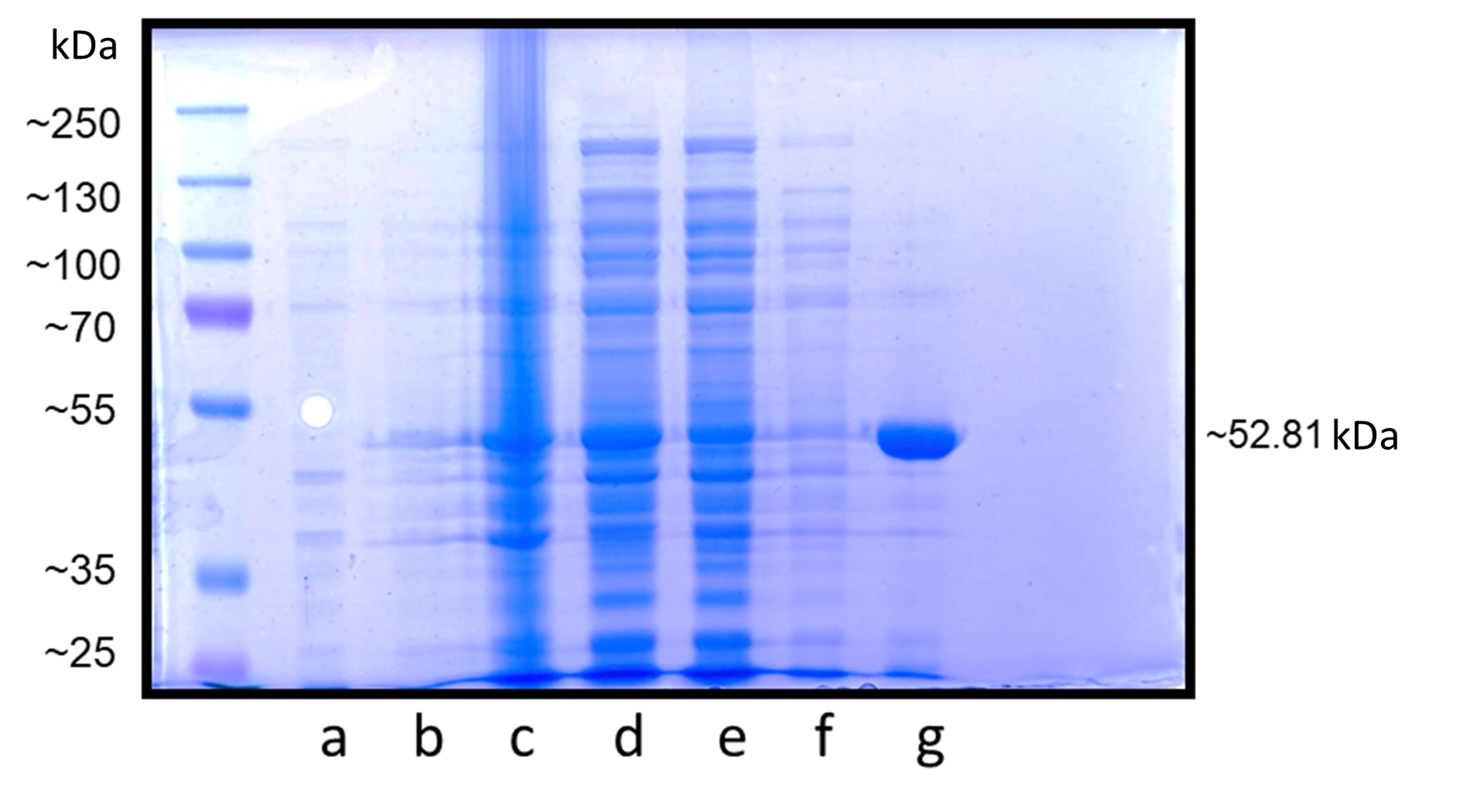

Supplement: Supplementary file 2 — Figurementary Figure 2. SDS‐PAGE of Iat4 expression and purification. (a) Non‐induced cells, (b) IPTG‐induced cells, (c) Cell lysis, (d) Soluble fraction, (e) Flow through, (f) Wash, (g) Purified extract. [file JPI-77-e70053-s001.png]

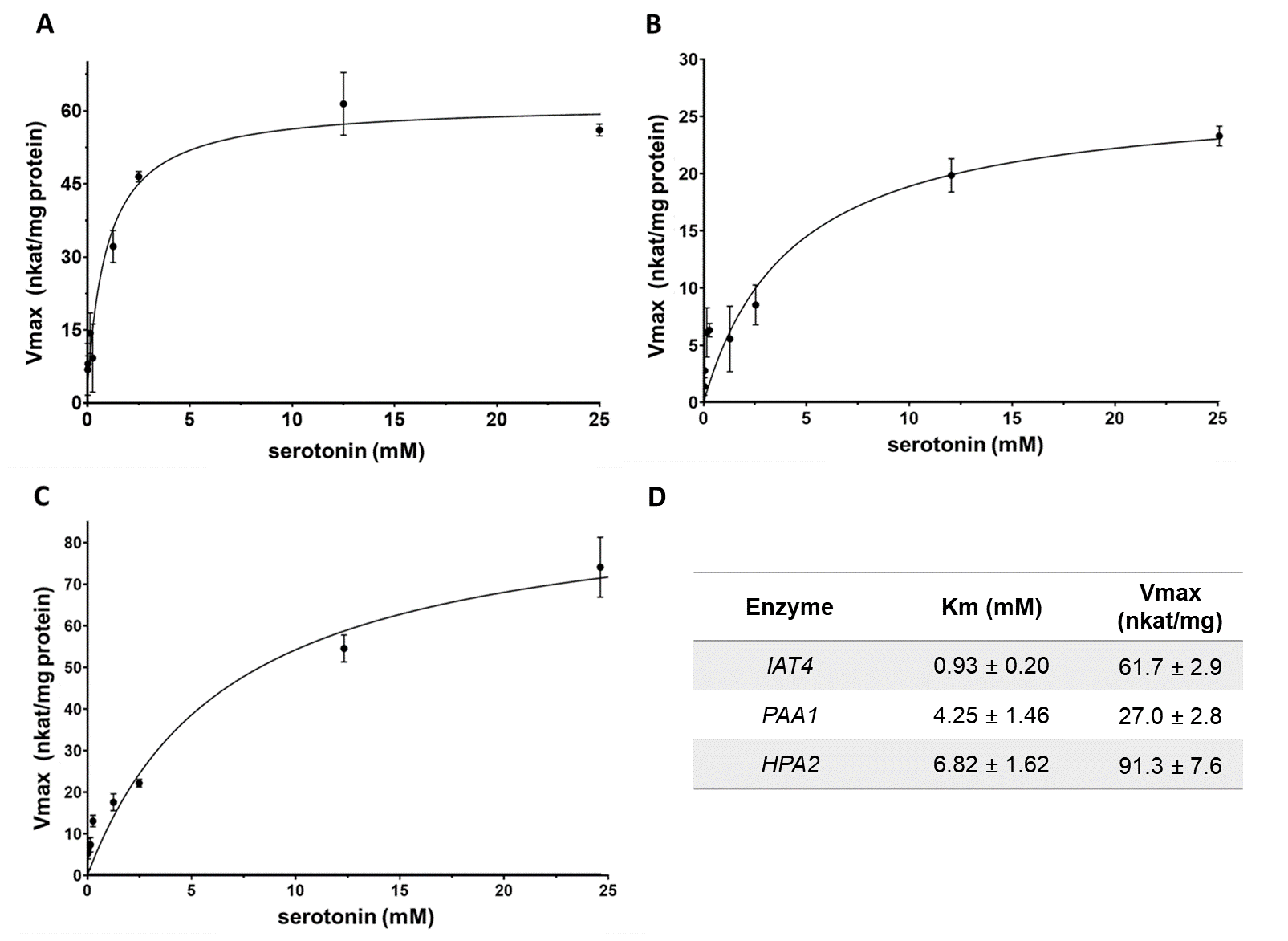

Supplement: Supplementary file 3 — Figurementary Figure 3. Michaelis–Menten enzyme kinetics for the enzymes. (A) IAT4 (B) PAA1, and (C) HPA2, using serotonin as substrate. (D) Table with the values of the affinity constant (Km ) and the maximum velocity (Vmax). Values are expressed as mean ± SD. All kinetics were performed with 7 points performed in triplicate. Values were fitted to Michaelis–Menten model, using R 2 as goodness of fit (PAA1: R² = 0.825; HPA2: R² = 0.939; IAT4: R² = 0.924). [file JPI-77-e70053-s004.png]

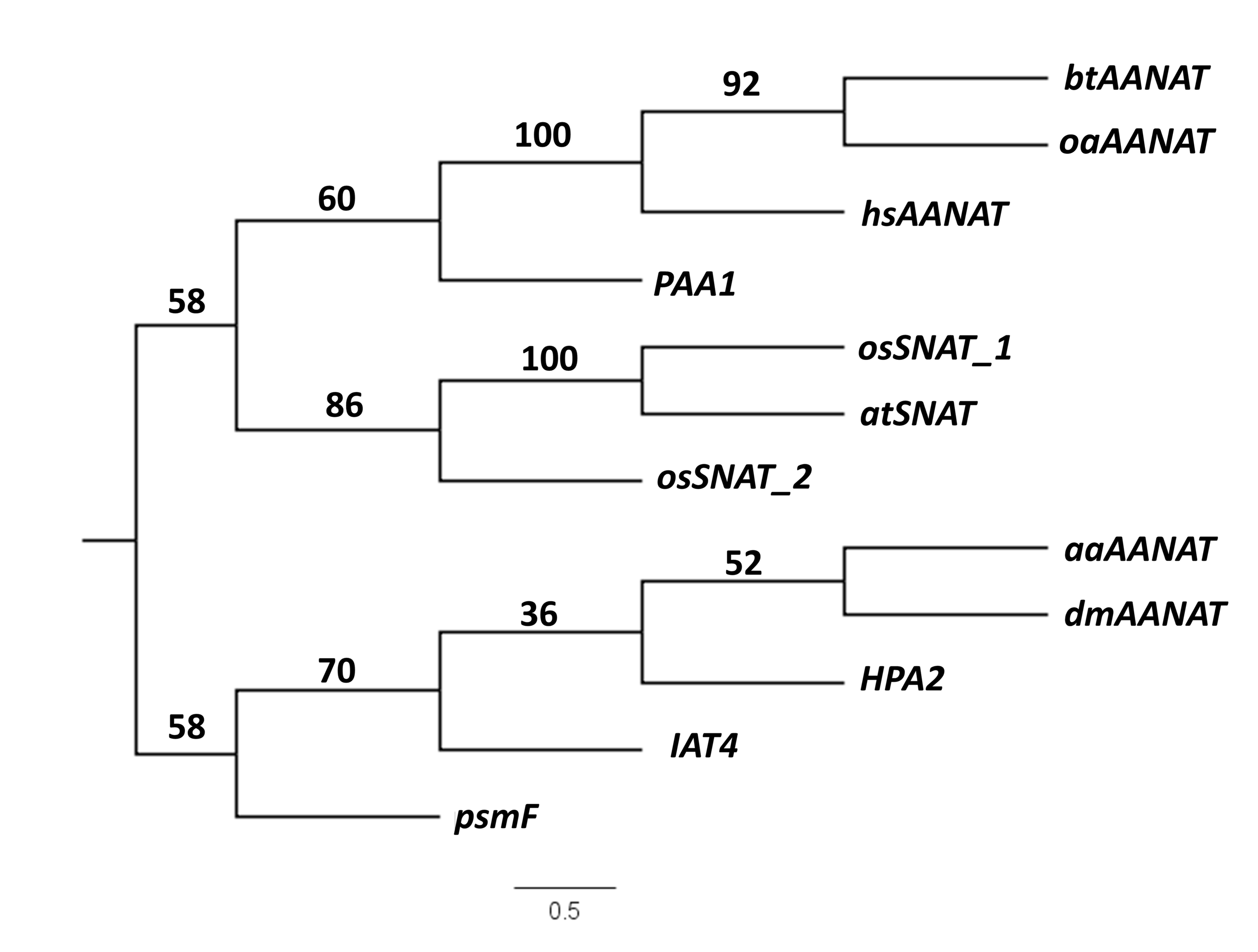

Supplement: Supplementary file 4 — Figurementary Figure 4. Phylogenetic tree of N‐acetyltransferases (NAT). The phylogenetic tree was constructed using Seaview based on the amino acid sequences of some representative organisms and yeast proteins candidates, being represented by their gene name: IAT4, HPA2, PAA1 (from S. cerevisiae), Bos taurus btAANAT, Ovis aries oaAANAT, Homo sapiens hsAANAT, Oryza sativa osSNAT (Version 1 and 2), Arabidopsis thaliana SNAT, Aedes aegypti AANAT, Drosophila melanogaster AANAT, psmF (from Streptomyces griseofuscus). Phylogenetic analysis was performed by using the Parsimony algorithm. The parameters were “Trees‐Parsimony‐Bootstrap method”. The number of Bootstrap replications was 1000, and ignore all gap sites was employed. [file JPI-77-e70053-s006.png]
