# Supplementary material for: IAT4, a New Indolamine N‐Acetyltransferase in Saccharomyces cerevisiae Involved in Melatonin Biosynthesis
Source: J Pineal Res. 2025 May 9;77(3):e70053. doi: 10.1111/jpi.70053 (PMC12063518; doi:10.1111/jpi.70053)
Supplement: Supplementary file 5 — Supplementary_Tables. [file JPI-77-e70053-s002.docx]

**Supplementary Tables**

**Supplementary Table 1**. List of the strains used in this study.

| **Strain name** | **Specie** | **Genomic modifications** | **Source** |  |
| --- | --- | --- | --- | --- |
| Rossetta (DE3) | *E. coli* | Genotype: F^-^ *ompT hsdS*_B_(r_B_^-^ m_B_^-^) *gal dcm* (DE3) pRARE (Cam^R^) | Novagen |  |
| Rossetta + pGEX 5x1 | *E. coli* | Rossetta (DE3) *with plasmid* pGEX 5x1 *empty (AmpR)* | This study |  |
| Rossetta + pGEX 5x1 IAT4 | *E. coli* | Rossetta (DE3) *with plasmid* pGEX 5x1 *IAT4 (AmpR)* | This study |  |
| Rossetta + pGEX 5x1 PAA1 | *E. coli* | Rossetta (DE3) *with plasmid* pGEX 5x1 *PAA1 (AmpR)* | This study |  |
| Rossetta + pGEX 5x1 AANAT | *E. coli* | Rossetta (DE3) *with plasmid* pGEX 5x1 *AANAT (AmpR)* | This study |  |
| BY4743 | *S. cerevisiae* | MATa/α *his3∆1/his3∆1* *leu2∆0*/*leu2∆0* *met15∆0*/*MET15* *LYS2*/*lys2∆0* *ura3∆0*/*ura3∆0* | Euroscarf |  |
| BY + pAP001 | *S.cerevisiae* | *BY4743 with plasmid* pAP001 *empty (URA3)* | This study |  |
| BY + pAP001 IAT4 | *S.cerevisiae* | *BY4743 with plasmid* pAP001 *IAT4 (URA3)* | This study |  |
| BY + pAP001 PAA1 | *S.cerevisiae* | *BY4743 with plasmid* pAP001 *PAA1 (URA3)* | This study | |
| BY + pAP001 AANAT | *S.cerevisiae* | BY4743 with plasmid pAP001 *AANAT* (*URA3*) | This study | |
| BS2 | *S.cerevisiae* | BY4743; TEF1p::*Cs*TDC; PGK1p::*Os*T5H, *LEU2, HIS3* | (Planells-Cárcel et al. 2025) | |
| BS2 + pCfB2988 | *S.cerevisiae* | *BS2 with linear plasmid* pCfB2988 *empty integrated (URA3)* | This study | |
| BS2 + pCfB2988 IAT4 | *S.cerevisiae* | *BS2 with linear plasmid* pCfB2988 *IAT4 integrated (URA3)* | This study | |
| BS2 + p426gpd | *S.cerevisiae* | *BS2 with plasmid p426GPD empty (URA3)* | This study | |
| BS2 + p426gpd IAT4 | *S.cerevisiae* | *BS2 with plasmid p426GPD IAT4 (URA3)* | This study | |

**Supplementary Table 2**. List of the plasmids used in this study.

| **Plasmid name** | **Description** | **Bacterial Marker** | **Yeast Marker** | **Source** |
| --- | --- | --- | --- | --- |
| pGEX-5X-1 | Taq promoter-GST-Multiple cloning site (MCS) | Amp | - | GE Healthcare |
| pGEX-5X-1 IAT4 | Taq promoter-GST-*IAT4* | Amp | - | This study |
| pGEX-5X-1 PAA1 | Taq promoter-GST-*PAA1* | Amp | - | This study |
| pGEX-5X-1 AANAT | Taq promoter-GST-*btAANAT* | Amp | - |  |
| pAP001 | Multicopy vector with galactose induction expression system (*GAL1* promoter) | Amp | URA3 | This study |
| pAP001 IAT4 | pAP001 with *IAT4* gen | Amp | URA3 | This study |
| pAP001 PAA1 | pAP001 with *PAA1* gen | Amp | URA3 | This study |
| pAP001 AANAT | pAP001 with *btAANAT* gen | Amp | URA3 | This study |
| p426gpd | Multicopy vector (URA3 marker, GPD promoter) | Amp | URA3 | (Mumberg et al., 1995) |
| pCfB2988 | Integrative vector for multiple integrations at sites sharing homology with Ty1Cons (Kl.URA3-deg marker) | Amp | URA3 | (Maury et al., 2016) |
| p426gpd IAT4 | p426GPD with *IAT4* | Amp | URA3 | This study |
| pCfB2988 IAT4 | pCfB2988 with *IAT4* | Amp | URA3 | This study |

**Supplementary Table 3**. List of the oligonucleotides used in this study.

| **Primer name** | **Sequence 5’→3’** |
| --- | --- |
| IAT4 F BamHI | GTCGTGGGATCCTTATGTCCTTCGAGAACAAACT |
| IAT4 R XhoI | CCGCTCGAGCTACTCTTTTAAGACTTTATTACCGT |
| PAA1 F BamHI | AGGTCGTGGGATCCCCATGGCCTCCTCAAGTAGCA |
| PAA1 R XhoI | TGCGGCCGCTCGAGCTAGTTGTCGTATTCTTCCTTAAT |
| AANAT F BamHI | AGGTCGTGGGATCCCCATGAGCACCCCGAGCAT |
| AANAT R XhoI | TGCGGCCGCTCGAGTTAACGATCGCTATTACGACGCA |
| pGEX seq F | GGGCTGGCAAGCCACGTTTGGTG |
| pGEX seq R | CCGGGAGCTGCATGTGTCAGAGG |
| Check pAP001 F | ACCTCTATACTTTAACGTCAAGGAG |
| Check pAP001 R | ACCTGAGAAAGCAACCTGACC |
| GP2F (IAT4) | ATCTGTCAUAAAACAATGTCCTTCGAGAACAAACTG |
| GV2R (IAT4) | CACGCGAUCTACTCTTTTAAGACTTTATTACCGTC |
| ADH1_test_fw | GAAATTCGCTTATTTAGAAGTGTC |
| CYC1_test_rv | CTCCTTCCTTTTCGGTTAGAG |
| GPD_test_fw | CGGTAGGTATTGATTGTAATTCTG |

**Supplementary Table 4**. Information on the pAP001 plasmid and the primers used for the construction of various parts.

| **Lenght** | **4963pb** | **Primers** | **Sequence 5’-3’** |
| --- | --- | --- | --- |
| Part type 1: | *pGAL1* (derivated from pYTK030) | MoClo Multicloning p426_F (type 2) | GCATCGTCTCATCGGTCTCAAACGCTAGAACTAGTGGATCCCCC |
| Part type 2-3: | Multicloning site derivated from p426gpd plasmid | MoClo Multicloning p426_R (type 3) | ATGCCGTCTCAGGTCTCAGGATTGACTCGAGGTCGACGGTAT |
| Part type 4: | *tADH1* (pYTK053) | MoClo pGAL1_F (type 1) | GCATCGTCTCATCGGTCTCACCCTCCCCATTATCTTAGCCTAAAAAAACC |
| Part type 5: | ConR (pYTK067) | MoClo pGAL1_R (type 1) | ATGCCGTCTCAGGTCTCACGTTCTTATAGTTTTTTCTCCTTGACGTTA |
| Part type 6: | *URA3* Marker (pYTK074) |  |  |
| Part type 7: | 2micron replication origin (pYTK082) |  |  |
| Part type 8: | AmpR marker and OriE from *E.coli*(pYTK083) |  |  |

**Supplementary Table 5**. Information of sequence for the new plasmid pAP001.

| >pAP001_sequence |
| --- |
| CCCCATTATCTTAGCCTAAAAAAACCttctctttggaactttcagtaatacgcttaactgctcattgctatattgaagtacggattagaagccgccgagcgggcgacagccctccgacggatgactctcctccgtgcgtcctcgtcatcaccggtcgcgttcctgaaacgcagatgtgcctcgcgccgcactgctccgaacaataaagattctacaatactagcttttatggttatgaagaggaaaaattggcagtaacctggccccacaaaccttcaaattaacgaatcaaattaacaaccataggatgataatgcgattagttttttagccttatttctggggtaattaatcagcgaagcgatgatttttgatctattaacagatatataaatggaaaagctgcataaccactttaactaatactttcaacattttcagtttgtattacttcttattcaaatgtcataaaagtatcaacaaaaaattgttaatatacctctatacttTAACGTCAAGGAGAAAAAACTATAAGaacgCTAGAACTAGTGGATCCCCCgggctgcaggaattcgatatcaagcttatcgATACCGTCGACCTCGAGTCAatcctaactcgaggcgaatttcttatgatttatgatttttattattaaataagttataaaaaaaataagtgtatacaaattttaaagtgactcttaggttttaaaacgaaaattcttattcttgagtaactctttcctgtaggtcaggttgctttctcaggtatagcatgaggtcgctcttattgaccacacctctaccggcatgccgagcaaatgcctgcaaatcgctccccatttcgctgccaatgagacgacggggtcatcacggctcatcatgcgccaaacaaatgtgtgcaatacacgctcggatgactgcatgatgaccgcactgactggggacagcagatccacctaagcctgtgagagaagcagacacccgacagatcaaggcagttaactagtgcactgcagtacacggtttccttgaaatttttttgattcggtaatctccgaacagaaggaagaacgaaggaaggagcacagacttagattggtatatatacgcatatgtagtgttgaagaaacatgaaattgcccagtattcttaacccaactgcacagaacaaaaacgtgcaggaaacgaagataaatcatgtcgaaagctacatataaggaacgtgctgctactcatcctagtcctgttgctgccaagctatttaatatcatgcacgaaaagcaaacaaacttgtgtgcttcattggatgttcgtaccaccaaggaattactggagttagttgaagcattaggtcccaaaatttgtttactaaaaacacatgtggatatcttgactgatttttccatggagggcacagttaagccgctaaaggcattatccgccaagtacaattttttactcttcgaggacagaaaatttgctgacattggtaatacagtcaaattgcagtactctgcgggtgtatacagaatagcagaatgggcagacattacgaatgcacacggtgtggtgggcccaggtattgttagcggtttgaagcaggcggcagaagaagtaacaaaggaacctagaggccttttgatgttagcagaattgtcatgcaagggctccctatctactggagaatatactaagggtactgttgacattgcgaagagcgacaaagattttgttatcggctttattgctcaaagagacatgggtggaagagatgaaggttacgattggttgattatgacacccggtgtgggtttagatgacaagggagatgcattgggtcaacagtatagaaccgtggatgatgtggtttctacaggatctgacattattattgttggaagaggactatttgcaaagggaagggatgctaaggtagagggtgaacgttacagaaaagcaggctgggaagcatatttgagaagatgcggccagcaaaactaaaaaactgtattataagtaaatgcatgtatactaaactcacaaattagagcttcaatttaattatatcagttattacccgagtaacgaagcatctgtgcttcattttgtagaacaaaaatgcaacgcgagagcgctaatttttcaaacaaagaatctgagctgcatttttacagaacagaaatgcaacgcgaaagcgctattttaccaacgaagaatctgtgcttcatttttgtaaaacaaaaatgcaacgcgagagcgctaatttttcaaacaaagaatctgagctgcatttttacagaacagaaatgcaacgcgagagcgctattttaccaacaaagaatctatacttcttttttgttctacaaaaatgcatcccgagagcgctatttttctaacaaagcatcttagattactttttttctcctttgtgcgctctataatgcagtctcttgataactttttgcactgtaggtccgttaaggttagaagaaggctactttggtgtctattttctcttccataaaaaaagcctgactccacttcccgcgtttactgattactagcgaagctgcgggtgcattttttcaagataaaggcatccccgattatattctataccgatgtggattgcgcatactttgtgaacagaaagtgatagcgttgatgattcttcattggtcagaaaattatgaacggtttcttctattttgtctctatatactacgtataggaaatgtttacattttcgtattgttttcgattcactctatgaatagttcttactacaatttttttgtctaaagagtaatactagagataaacataaaaaatgtagaggtcgagtttagatgcaagttcaaggagcgaaaggtggatgggtaggttatatagggatatagcacagagatatatagcaaagagatacttttgagcaatgtttgtggaagcggtattcgcaatattttagtagctcgttacagtccggtgcgtttttggttttttgaaagtgcgtcatcagagcgcttttggttttcaaaagcgctctgaagttcctatactttctagctagagaataggaacttcccgagcggccgcgattatcaaaaaggatcttcacctagatccttttaaattaaaaatgaagttttaaatcaatctaaagtatatatgagtaaacttggtctgacagttaccaatgcttaatcagtgaggcacctatctcagcgatctgtctatttcgttcatccatagttgcctgactccccgtcgtgtagataactacgatacgggagggcttaccatctggccccagtgctgcaatgataccgcgggacccacgctcaccggctccagatttatcagcaataaaccagccagccggaagggccgagcgcagaagtggtcctgcaactttatccgcctccatccagtctattaattgttgccgggaagctagagtaagtagttcgccagttaatagtttgcgcaacgttgttgccattgctacaggcatcgtggtgtcacgctcgtcgtttggtatggcttcattcagctccggttcccaacgatcaaggcgagttacatgatcccccatgttgtgcaaaaaagcggttagctccttcggtcctccgatcgttgtcagaagtaagttggccgcagtgttatcactcatggttatggcagcactgcataattctcttactgtcatgccatccgtaagatgcttttctgtgactggtgagtactcaaccaagtcattctgagaatagtgtatgcggcgaccgagttgctcttgcccggcgtcaatacgggataataccgcgccacatagcagaactttaaaagtgctcatcattggaaaacgttcttcggggcgaaaactctcaaggatcttaccgctgttgagatccagttcgatgtaacccactcgtgcacccaactgatcttcagcatcttttactttcaccagcgtttctgggtgagcaaaaacaggaaggcaaaatgccgcaaaaaagggaataagggcgacacggaaatgttgaatactcatactcttcctttttcaatattattgaagcatttatcagggttattgtctcatgagcggatacatatttgaatgtatttagaaaaataaacaaataggggttccgcgcacatttccccgaaaagtgccacctgtcatgaccaaaatcccttaacgtgagttttcgttccactgagcgtcagaccccgtagaaaagatcaaaggatcttcttgagatcctttttttctgcgcgtaatctgctgcttgcaaacaaaaaaaccaccgctaccagcggtggtttgtttgccggatcaagagctaccaactctttttccgaaggtaactggcttcagcagagcgcagataccaaatactgttcttctagtgtagccgtagttaggccaccacttcaagaactctgtagcaccgcctacatacctcgctctgctaatcctgttaccagtggctgctgccagtggcgataagtcgtgtcttaccgggttggactcaagacgatagttaccggataaggcgcagcggtcgggctgaacggggggttcgtgcacacagcccagcttggagcgaacgacctacaccgaactgagatacctacagcgtgagctatgagaaagcgccacgcttcccgaagggagaaaggcggacaggtatccggtaagcggcagggtcggaacaggagagcgcacgagggagcttccagggggaaacgcctggtatctttatagtcctgtcgggtttcgccacctctgacttgagcgtcgatttttgtgatgctcgtcaggggggcggagcctatggaaaaacgccagcaacgcggcctttttacggttcctggccttttgctggccttttgctcacatgttctttcctgcgttatcccctgattctgtggataaccgtgcggccgcccct |
